# *Alternaria arborescens* and *A. italica* Causing Leaf Blotch on *Celtis julianae* in China

**DOI:** 10.3390/plants12173113

**Published:** 2023-08-30

**Authors:** Yang-Chun-Zi Liao, Yi-Jia Cao, Yu Wan, Hui Li, De-Wei Li, Li-Hua Zhu

**Affiliations:** 1 College of Forestry, Nanjing Forestry University, Nanjing 210037, Chinayjcao@njfu.edu.cn (Y.-J.C.); wanyu@njfu.edu.cn (Y.W.); lhui@njfu.edu.cn (H.L.); 2 Co-Innovation Center for Sustainable Forestry in Southern China, Nanjing Forestry University, Nanjing 210037, China; 3 The Connecticut Agricultural Experiment Station Valley Laboratory, Windsor, CT 06095, USA

**Keywords:** *Celtis julianae*, *Alternaria*, multi-locus phylogeny, new disease, identification

## Abstract

*Celtis julianae* Schneid. is widely planted as a versatile tree species with ecological and economic significance. In September 2022, a leaf blotch disease of *C. julianae* was observed in Nanjing, Jiangsu, China, with an infection incidence of 63%. The disease led to severe early defoliation, significantly affecting the ornamental and ecological value of the host tree. The accurate identification of pathogens is imperative to conducting further research and advancing disease control. Koch’s postulates confirmed that the fungal isolates (B1–B9) were pathogenic to *C. julianae*. The morphology of the characteristics of the pathogen matched those of *Alternaria* spp. The internal transcribed spacer region (ITS), large subunit (*LSU*) and small subunit (*SSU*) regions of rRNA, glyceraldehyde-3-phosphate dehydrogenase (*GAPDH*), *Alternaria* major allergen gene (*Alt a 1*), RNA polymerase second largest subunit (*RPB2*), and portions of translation elongation factor 1-alpha (*TEF1-α*) genes were sequenced. Based on multi-locus phylogenetic analyses and morphology, the pathogenic fungi were identified as *Alternaria arborescens* and *A. italica*. The findings provided useful information for disease management and enhanced the understanding of *Alternaria* species diversity in China. This is the first report of *A. arborescens* and *A. italica* causing leaf blotch of *C. julianae* in China and worldwide.

## 1. Introduction

*Celtis julianae* Schneid. (Ulmaceae) is a large deciduous tree that can reach a height of over 25 m. It serves as a valuable landscaping and shelter tree species due to its ability to withstand soot and toxic gases [1]. Additionally, its kernels are used as raw material for soap and lubricating oil production due to their high oil content. The numerous hairs on leaf surfaces enable it to trap airborne particulates, contributing to air purification [2]. In September 2022, a leaf blotch disease was observed on *C. julianae* at Nanjing Forestry University (118°48′26″ E, 32°4′52″ N). The disease led to early defoliation in severe cases, resulting in a reduction of its ornamental and ecological value.

The *Alternaria* genus comprises approximately ca. 382 species separated into 29 sections [3,4,5,6]. *Alternaria* species are crucial invasive pathogen that can colonize a wide range of hosts, including various plants in the phyllosphere, and animals, including humans [3,7,8,9,10]. Over 4000 monocotyledonous and dicotyledonous plant species are affected by *Alternaria* spp. [3,6,11,12,13]. For example, *A. alternata* (Fr.) Keissler is known to cause leaf spots on *Prunus salicina* [14]. In Oman, several species of *Alternaria* are associated with leaf spots on date palm and wheat produce, leading to reduced market value and significant economic losses [15]. Additionally, *A. arborescens* E.G. Simmons has been reported to cause leaf spots on *Pereskia aculeata* in Brazil [16]. In addition, the conidia of *Alternaria* are the most common airborne allergens and have been determined to be significant triggers of allergic rhinitis and allergic asthma [17,18]. Furthermore, *Alternaria* species have emerged as important human invasive pathogens in immune-compromised patients [19,20]. Thus, the *Alternaria* species deserves further studies.

The traditional identification of plant pathogenic fungi has mainly relied solely on morphological characteristics and host association. However, when relying solely on morphological characteristics, such as conidia, conidiophores, conidiogenous cells, and fruiting body, the pathogenic fungi may not be accurately classified and determined. Phylogenetic analyses have been widely applied for decades, leading to the discovery of numerous new species [21,22,23]. Initially, the internal transcribed spacer region (ITS) was commonly used for taxonomic purposes. However, studies have pointed out the limitations of using ITS sequence data [24,25,26]. In many fungal genera, the ITS locus only resolves taxa to the genus level due to the intraspecific and even intragenomic polymorphisms commonly observed [27,28]. Moreover, a substantial percentage of ITS sequences in GenBank are derived from misidentified specimens or cultures [29,30]. As a result, secondary DNA barcodes have been proposed for various genera of plant pathogenic fungi [31,32,33]. Nowadays, multi-locus phylogenetic analysis is considered more appliable and reliable in fungal taxonomy [34].

Similarly, *Alternaria* species were traditionally classified based on morphological characteristics of their reproductive structures and sporulation patterns under various conditions [35]. *Alternaria* spp. are dematiaceous fungi, exhibiting grey-olive/brown colonies. The conidia of *Alternaria* are dictyospores. These can either be found alone or in chains [36]. However, the subtle differences between *Alternaria* spp. and the morphological variation under different conditions can make identification based on solely on morphological characteristics challenging. With the advent of molecular analyses, researchers have examined phylogenetic relationships among *Alternaria* species. Lawrence et al. [12,18] first determined 27 sections in *Alternaria* through multi-locus phylogeny. Woudenberg et al. [6] used six loci to establish 24 sections, including 16 newly described sections. By combining seven loci, a consensus phylogeny was generated. Ghafri et al. [37] introduced *Alternaria* section *Omanenses* in 2019, and Gannibal et al. [38] introduced *Alternaria* section *Helianthiinficiens* in 2022, bring the total number of *Alternaria* species to 29 sections. Currently, a clear and stable phylogenetic classification has been established at the species level among *Alternaria* spp., providing a reliable basis for this study.

The objectives of this study were (1) to isolate and identify the pathogen of leaf blotch on *C. julianae* according to morphological characteristics and multilocus phylogenetic analyses, and (2) to confirm the pathogenicity of the isolates on *C. julianae*.

## 2. Results

### 2.1. Disease Symptoms and Fungal Isolations

**Figure 1:**
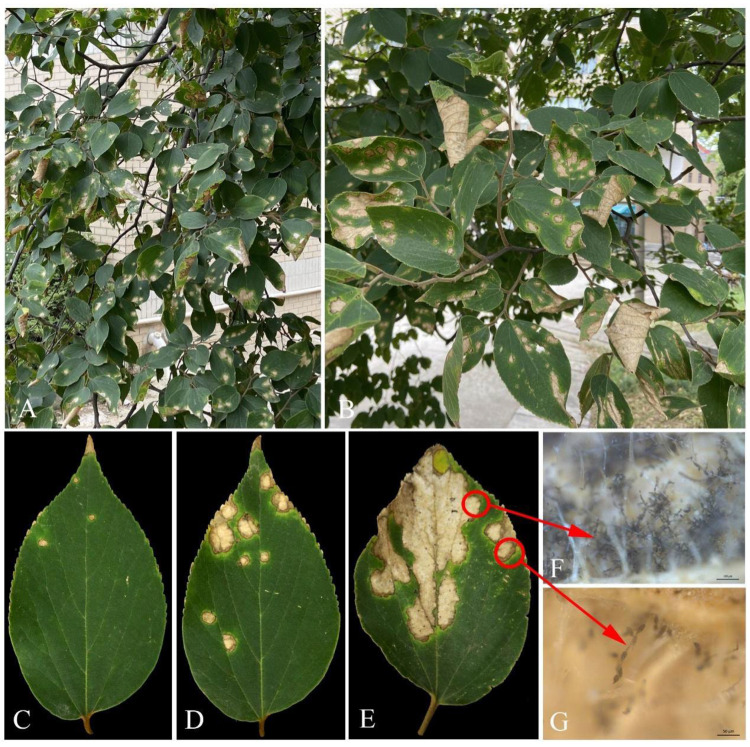
Symptoms of leaf blotch on *Celtis julianae* in the field. (**A**,**B**) Symptoms on diseased leaves; (**C**–**E**) diseased leaves on early stage, middle stage, and later stage, respectively; (**F**,**G**) conidial chains on a lesion of a leaf, scale bars: F = 100 μm, G = 50 μm.

The leaf blotch disease was observed on *C. julianae* at the campus of Nanjing Forestry University, China, with an incidence of 63% (63/100 plants). On average, 80% of the leaves per tree showed the presence of this leaf blotch disease (Figure 1A,B). The symptoms initially appeared as small, light brown spots. Measuring 1–2 mm in diameter, they were surrounded by yellow halos (Figure 1C). Subsequently, the spots gradually expanded into irregular necrotic blotches with dark brown borders, and the halos developed around the dark brown borders, leading to an increase in the size of the blotches over time (Figure 1D). Eventually, the blotches coalesced into large necrotic areas, resulting in leaf shrinkage, wrinkling, and collapse (Figure 1E). Numerous brown conidial chains were observed on the lesions of infected leaves (Figure 1F,G).

Diseased leaves were collected twice for isolating fungal pathogens in September and in October 2022, respectively. In the September experiment, three types of colonies were found with a frequency of 63%, 24%, and 13%, respectively. Similarly, in the October experiment, three types of colonies were found with a frequency of 54%, 38%, and 8%, respectively. According to the ITS sequence alignment, the three types of colonies belonged to the genera *Nigrospora*, *Alternaria* and *Nothophoma*, respectively (Table 1).

### 2.2. Pathogenicity Tests

**Figure 2:**
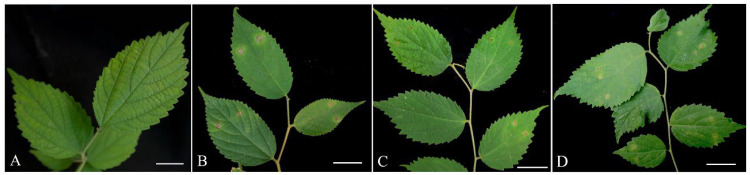
Pathogenicity of *Alternaria* isolates (B1, B2 and B3) on *Celtis julianae*. (**A**) No symptoms showing on the leaves from control plants 7 days after inoculation with sterile water. (**B**–**D**) Symptoms on leaves 7 days after inoculation with conidial suspensions of B1 (**B**), B2 (**C**), and B3 (**D**), respectively. Scale bars: (**A**–**D**) = 2 cm.

Five days post-inoculation in vitro, all detached leaves inoculated with nine isolates (B1–B9) of *Alternaria* spp. appeared to display brown spot symptoms at the inoculation sites, whereas leaves inoculated with *Nigrospora* sp., *Nothophoma* sp. and control leaves did not show any symptoms. Subsequently, in vivo experiments were conducted that used three representative fungal isolates (B1, B2 and B3) of the *Alternaria* species to inoculate healthy *C. julianae* seedlings. Seven days after inoculation, the inoculated leaves appeared to be brown spots with yellow halos, resembling the early symptoms observed on the leaves infected in the field. In contrast, the control leaves remained healthy (Figure 2). The same fungus was re-isolated from the lesions, and no other fungi were isolated from the control leaves. This fulfills Koch’s postulates, confirming that isolates B1, B2 and B3 are the causal agents of leaf blotch on *C. julianae*.

### 2.3. Multigene Phylogenetic Analyses

**Figure 3:**
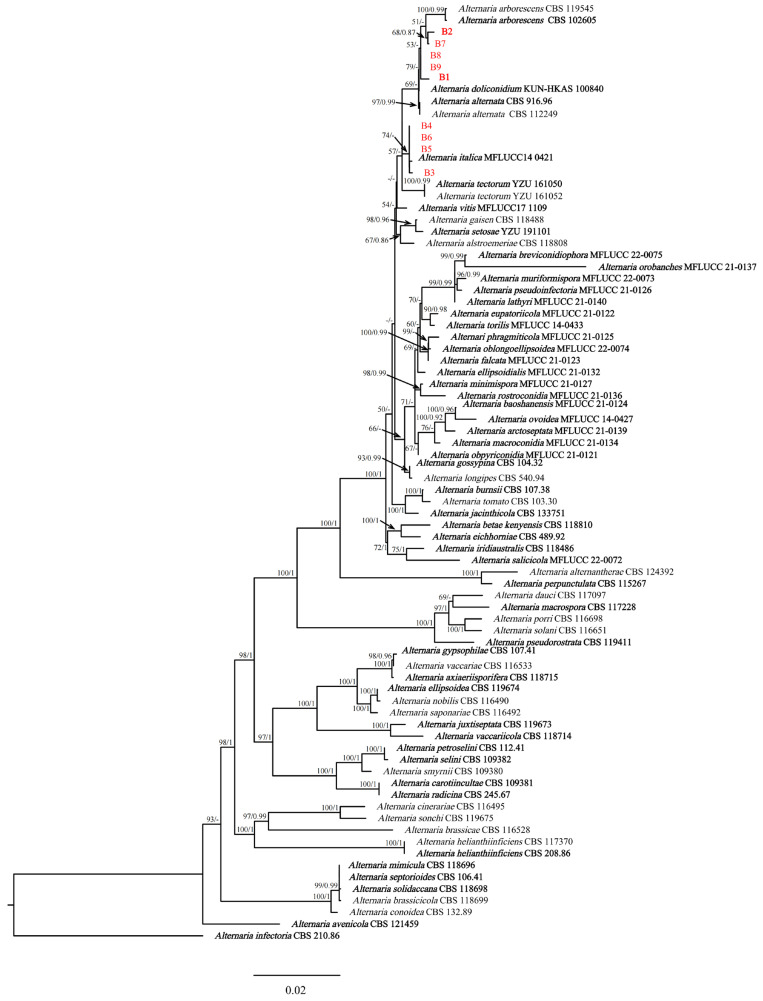
Phylogenetic relationship of *Alternaria arborescens* (B1, B2, B7, B8 and B9), and *A. italica* (B3, B4, B5 and B6) with related taxa derived from maximum-likelihood (ML) and Bayesian posterior probability analysis using concatenated ITS, *LSU*, *SSU*, *GAPDH*, *Alt a 1*, *RPB2* and *TEF1-α* sequences of *Alternaria* spp., with *Alternaria infectoria* (CBS 210.86) as the outgroup. RA × ML bootstrap support values (ML ≥ 70) and Bayesian posterior probability values (PP ≥ 0.70) were shown at the nodes (ML/PP). Bar = 0.02 substitution per nucleotide position. The ex-type strains are in bold.

Phylogenetic analyses performed using maximum-likelihood and Bayesian inference techniques placed the four isolates (B3, B4, B5, and B6) in the same cluster with *Alternaria italica* J.F. Li, Camporesi & K.D. Hyde (extype: MFLUCC 14-0421T). Meanwhile, there were five isolates (B1, B2, B7, B8, and B9) in the same cluster with *A. arborescens* (extype: CBS 102605) (Figure 3). The phylogenetic trees generated using ML and BI methods showed consistent topology. Based on the phylogenetic analyses conducted using the concatenated sequences of seven genes/regions (ITS, *LSU*, *SSU*, *GAPDH*, *Alt a 1*, *RPB2* and *TEF1-α*), B1, B2, B7, B8, and B9 were identified as *A. arborescens*, while B3, B4, B5, and B6 were determined to be *A. italica*.

### 2.4. Morphology and Taxonomy

**Figure 4:**
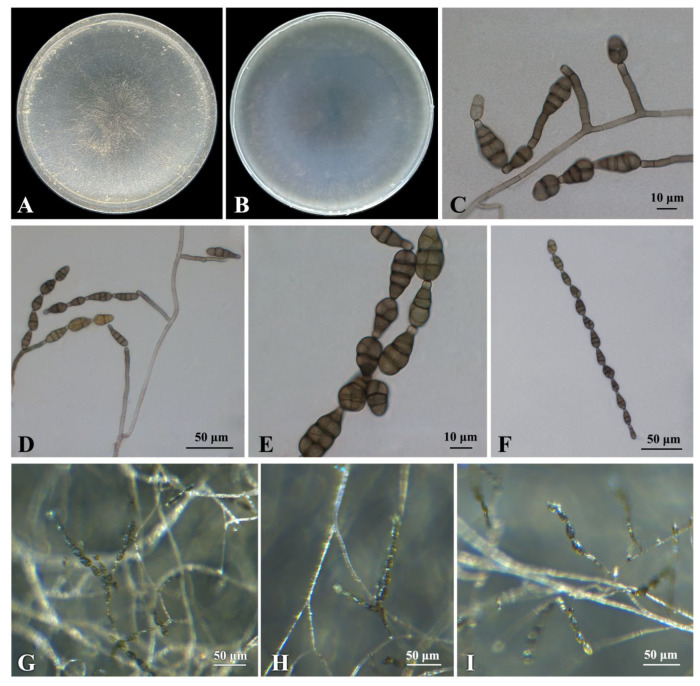
Morphological characteristics of *Alternaria arborescens* (isolate B1). (**A**) Front and (**B**) reverse view of 7-day-old colony on PCA; (**C**,**D**) conidiophores and conidia; (**E**) conidia; (**F**) conidial chains; (**G**–**I**) mycelia, conidiophores and conidial chains on PCA under a Zeiss stereo microscope.

***Alternaria arborescens*** E.G. Simmons (Figure 4)

**Culture characteristics:** On potato–carrot agar (PCA) and V8 agar (V-8), colonies of isolate B1 were circular, flat, and granulated with undulating edges. The colony was grayish green, and the reverse side was greenish brown (Figure 4A,B).

**Description:** Sexual morph not observed. Under the Zeiss Axio Imager A2m microscope (Carl Zeiss AG, Oberkochen Germany), the hyphae were hyaline to light brown, septate, and (3.0−)3.7 − 5.3(−7.1) μm (mean ± SD = 4.5 ± 0.8 μm, n = 30) wide. Conidiophores were solitary, dark brown, straight or curved, 2–8 septa, and variable in length, (32.7−)41.5 − 94.3(−121.6) × (3.2−)3.7 − 4.7(−5.3) μm (mean ± SD = 67.9 ± 26.4 × 4.2 ± 0.5 μm, n = 30) (Figure 4C,D). Conidiogenous cells (5.5−)6.2 − 9.2(−11.8) × (3.4−)4.0 − 4.8(−5.0) μm (mean ± SD = 7.7 ± 1.5 × 4.4 ± 0.4 μm, n = 30). Conidia were oval or obclavate, brown to dark brown, with 1–4 transverse septa and 1–4 longitudinal or oblique septa, constricted at the septa, (19.7−)23.6 − 28.4(−34.3) × (9.8−)11.0 − 12.8(−13.7) μm (mean ± SD = 26.0 ± 2.4 × 11.9 ± 0.9 μm, n = 30) (Figure 4E). The beaks (2.7−)3.3 − 5.1(−6.4) × (2.8−)3.4 − 4.4(−4.9) μm (mean ± SD = 4.2 ± 0.9 × 3.9 ± 0.5 μm, n = 30). Conidial chains were simple or branched with 1–15 conidia. (Figure 4F).

**Specimens examined:** China, Jiangsu province, Nanjing city, isolated from leaves of *Celtis julianae*, 1 September 2022, Yijia Cao, cultures: CFCC 59038 (=B1), CFCC 59039 (=B2), B7, B8 and B9.

**Notes:** The phylogenetic analyses showed that five isolates (B1, B2, B7, B8 and B9) were in a clade with *A. arborescens* (Figure 3). The morphological features of the five isolates matched those of *A. arborescens* [39]. Based on the morphology and phylogeny, B1, B2, B7, B8 and B9 were identified as *A. arborescens*.

**Figure 5:**
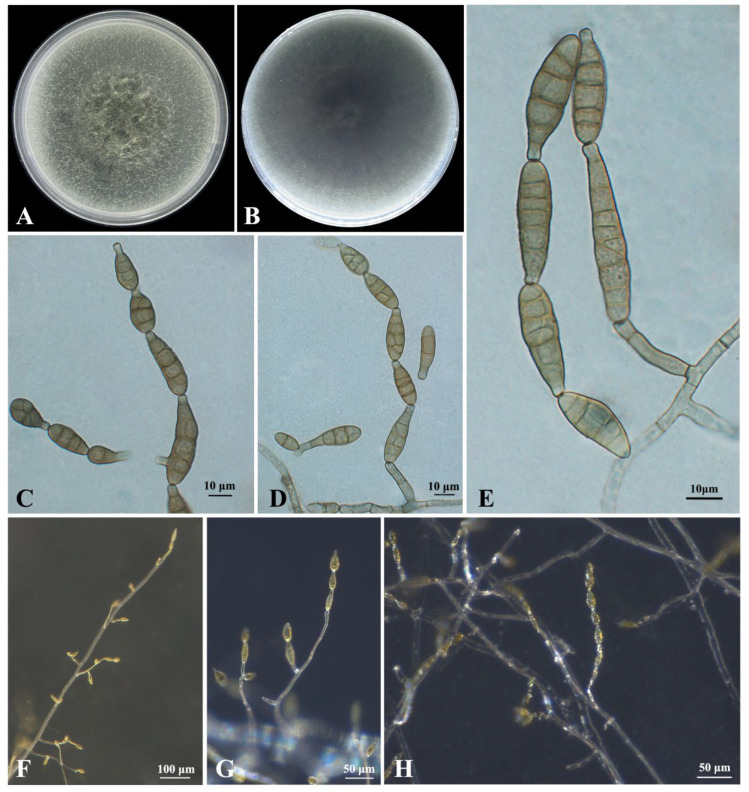
Morphological characteristics of *Alternaria italica* (isolate B3). (**A**) Front and (**B**) reverse view of 7-day-old colony on PCA. (**C**–**E**) Conidia, conidiophores and conidial chains. (**F**–**H**) Mycelia, conidiophores and conidial chains on PCA under a Zeiss stereo microscope.

***Alternaria italica*** J.F. Li, Camporesi & K.D. Hyde (Figure 5)

**Culture characteristics:** On PCA and V-8 media, the colony appears flat with gray margin. It is greyish green and cottony, covering the Petri dish after 7 days and showing abundant sporulation. The reverse side is dark greyish-green and radial (Figure 5A,B). After a week, the culture has increased the amount of white aerial mycelium in the center of the plate.

**Description:** Sexual morph not observed. The hyphae were colorless, hyaline to light brown, septate, (3.2−)3.6 − 5.0(−5.7) μm (mean ± SD = 4.3 ± 0.7 μm, n = 30) wide. Conidiophores macronematous, mononematous, flexuous or sigmoid, 0–5 septate, simple or branched, smooth, and hyaline to light brown, (13.5−)20.1 − 36.7(−46.2) × (3.1−)3.8 − 4.8(−5.3) μm (mean ± SD = 28.4 ± 8.3 × 4.3 ± 0.5 μm, n = 30) (Figure 5D,E). Lateral secondary conidiophores were observed but relatively uncommon. Conidiogenous cells were at the tip of conidiophores, (4.8−)5.8 − 8.0(−10.1) × (3.4−)3.8 − 4.8(−6.2) μm (mean ± SD = 6.9 ± 1.1 × 4.3 ± 0.5 μm, n = 30). Conidial chains were commonly single file, occasionally branched with 2–8 conidia (Figure 5C,D). Conidia dictyospores, pale brown to brown, variable in size and shape, but often obclavate to obpyriform, with up to 8 transverse and usually 0–2 longitudinal or oblique septa, slightly constricted at the septa, (27.7−)29.6 − 40.0(−51.3) × (7.6−)8.2 − 10.8(−13.1) μm (mean ± SD = 34.8 ± 5.2 × 9.5 ± 1.3 μm, n = 30) (Figure 5C). The apex of the conidia bears a beak, pale brown, most of the beaks are relatively short and well rounded, (3.1−)4.0 − 8.2(−12.2) × (3.1−)3.5 − 4.3(−5.1) μm (mean ± SD = 6.1 ± 2.1 × 3.9 ± 0.4 μm, n = 30).

**Specimens examined:** China, Jiangsu province, Nanjing city, isolated from leaves of *Celtis julianae*, 1 September 2022, Yijia Cao, cultures: CFCC 59359 (=B3), CFCC 59309 (=B4), CFCC 59310 (=B5), and CFCC 59311 (=B6).

**Notes:** The phylogenetic analyses showed that four isolates (B3, B4, B5 and B6) clustered together with *A. italica* (Figure 3). Morphological features of the four isolates matched those of *A. italica* [40]. Based on morphology and phylogeny, B3, B4, B5 and B6 were identified as *A. italica*.

## 3. Discussion

The genus *Alternaria* was first described by Nees von Esenbeck (1816), with *A. tenuis* as the type of species. Historically, the identification and classification of *Alternaria* species heavily relied on measurements and descriptions of morphological characteristics [41], complemented later by molecular phylogeny [42,43]. However, recent studies have challenged the morphological basis for the identification of some species in *Alternaria* [11,13,44]. Currently, the genus *Alternaria* contains 29 sections, and the main morphological characteristic of *Alternaria* sect. *Alternaria* is the production of short conidia in chains [6,18,45]. Woudenberg et al. [6] established species in *Alternaria* sect. *Alternaria* based on ITS. Subsequent whole-genome sequencing, transcriptome comparisons, and multi-gene sequencing further rearranged *Alternaria* sect. *Alternaria* into 11 phylogenetic species and one species complex [13]. Following a number of studies conducted by Li et al. [3,4], Cannibal et al. [45,46], Wanasinghe et al. [47], Jayawardena et al. [48] and Nishikawa et al. [49], currently over 89 species constitute the sect. *Alternaria*. Traditional morphological methods with molecular phylogeny are vital for identifying *Alternaria* species. In this study, a multi-locus phylogeny based on a concatenated ITS, *LSU*, *SSU*, *GAPDH*, *Alt a 1*, *RPB2* and *TEF1-α* sequences, combined with morphological characteristics, revealed two known species, *A. arborescens* and *A. italica*, within the sect *Alternaria*. Additionally, our study also reported new host records for *A. arborescens* and *A. italica*. It is worth noting that many pathogenic fungal species are named after their hosts, and that the two *Alternaria* species identified in this study belong to the same section and is isolated from the same host. The similarities in their morphological features highlight the importance of taking cautious approaches in identifying and naming *Alternaria* species.

*Alternaria* is among the primary mycotoxigenic fungal genera found in cereals worldwide [50]. It can produce a variety of mycotoxins derived from secondary metabolism, with about 70 toxic metabolites described so far [35]. Some pathogenic *Alternaria* spp. utilize a diverse array of pathogenic toxins to infect plant tissues [51,52]. Certain species of *Alternaria* produce host-specific toxins (HSTs) that influence their pathogenicity and virulence. For example, the Japanese pear pathotype associated with *Alternaria* produces AK toxins I and II, which only exhibit toxicity in susceptible pear cultivars [35]. In addition, HSTs affect the taxonomy of the genus as fungal systematics include the analysis of secondary metabolites, providing information for use in species differentiation. At present, several researchers have employed chemotaxonomy and pathogenicity characteristics to assist with classification [53,54,55]. However, the method cannot be used independently for classification, because members within the same group may not share a common metabolite profile [50]. Therefore, the application of polyphasic approaches could provide strong support in fungal taxonomy, and further studies are warranted.

In our study, we observed that the isolation rates of *Nigrospora* spp. were consistently higher than those of *Alternaria* spp. in two independent fungal isolations. However, coexistence observation and pathogenicity testing showed that *Alternaria* spp. was the pathogen causing leaf blotching of *C. julianae*. Previous studies have indicated that species the of genus *Nigrospora* possess a strong saprobic nature and commonly occur as plant endophytes or saprobes on different hosts [56,57]. *Nigrospora* spp. may have more advantages in competing for nutrients in substrates compared to *Alternaria* spp., which can slow the growth of *Alternaria*. This could be the reason for the higher isolating rates of *Nigrospora* than those of *Alternaria* spp.

*Celtis julianae* is an important ornamental species mainly found in the temperate biome, with its native range overlaying the central and southern parts of China [58]. It has a high level of wind resistance due to its deep roots, making it an ideal species for embankment protection and water conservation [59]. Additionally, *C. julianae* helps maintain air humidity, reduce heat, and create various shadows with its large and dense leaves [60]. Economically, *C. julianae* has rich bark fiber, which can be used for making paper, and the core of its fruit can be used in oil and soap manufacture. In summary, *C. julianae* is a multi-functional species with ornamental, ecological and economic value. Currently, research on diseases affecting *C. julianae* is insufficient. Therefore, the investigation of leaf blotch on *C. julianae* and identification of pathogens have significance in providing the theoretical basis for reducing the economic and ecological losses caused by this disease.

## 4. Materials and Methods

### 4.1. Sampling and Isolation of Fungi

From September to October 2022, the isolation of fungi was conducted twice. Each time, 25 symptomatic leaves were collected from three infected plants on the campus of Nanjing Forestry University. The symptomatic leaves were first rinsed under running water and dried on sterilized filter paper. One-hundred small tissue (3 × 3 mm) samples were cut from lesion margins and surface-sterilized in 75% ethanol for 30 s, followed by the use of 1% NaClO for 90 s. The samples were then rinsed 3 times in sterile water, dried on sterilized filter paper, and finally plated onto potato dextrose agar (PDA) supplemented with ampicillin. The plates were incubated at 25 °C in an incubator, MIR-553 (Sanyo, Osaka Japan) [61]. After 3 days, the growing edges of the mycelium from the plant materials were cut and transferred to fresh PDA media to obtain a pure culture [62].

### 4.2. Pathogenicity Tests

To determine the pathogenicity of the isolates, experiments were conducted on both detached leaves and attached leaves. Healthy leaves from the field were collected, rinsed under tap-water for 15 min, and then wounded with sterile needles after drying on sterilized filter paper. For each type of test, two isolates were selected. For inoculation, plugs (5 mm diam.) were cut out from the growing edges of 5-day-old cultures and placed onto the wounds. The PDA plugs were used as controls. Five wounded leaves were treated with isolate and control plug. After inoculation, the leaves were placed into Petri dishes to maintain their humidity and kept at 25 °C in an incubator. Subsequently, nine representative fungal isolates (B1–B9) were selected from pathogenic species for in vivo inoculation with mycelial plugs. To confirm Koch’s postulates, further pathogenicity tests were performed by inoculating conidial suspensions on leaves of *C. julianae* seedlings that were wounded with a sterile needle. The leaves were inoculated with the three representative isolates (B1–B3) using 10 µL of conidial suspensions (10^6^ conidia/mL). Five plants were used for each treatment, and three leaves of each plant were inoculated. The control was treated with 10 µL of sterile water. All inoculated seedlings were covered with plastic bags, and sterilized water was sprayed into the bags daily to maintain a moist microclimate. All seedlings were kept in a growing chamber at 20 ± 2 °C/10 ± 2 °C (day/night) and observed regularly. Leaves that showed typical symptoms after the inoculation were used for re-isolations.

### 4.3. DNA Extraction and PCR Amplification

Nine isolates were cultured on potato dextrose agar (PDA), V8 agar (V-8) and potato–carrot agar (PCA) media at 25 °C in a constant temperature incubator with a 12/12 h light/dark cycle. Morphological identification was based on colony morphology and characteristics of conidia, conidiophores, conidiogenous cells, such as the shape and color of the colony, as well as the shape, size, color, septation, and presence of a beak in the conidia. A Zeiss Axio Imager A2m microscope (Carl Zeiss AG, Oberkochen Germany) and Zeiss stereo microscope (SteRo Discovery v20) were used to observe, describe, and measure conidia and other structures (n = 30).

### 4.4. Morphological Identification

For accurate pathogen identification, the genomic DNA was extracted using a modified CTAB method [63]. The internal transcribed spacer (ITS) [64], large subunit (*LSU*) and small subunit (*SSU*) regions of rRNA [64,65], glyceraldehyde-3-phosphate dehydrogenase (*GAPDH*) [66], *Alternaria* major allergen gene (*Alt a 1*) [67], RNA polymerase second largest subunit (*RPB2*) [68] and portions of translation elongation factor 1-alpha (*TEF1-α*) [69] genes/region were sequenced using primers ITS1/4, LR0R/LR05, NS1/NS4, GPD1/GPD2, Alt-for/Alt-rev, RPB2-5F2/RPB2-7cR and EF1-728F/EF1-986R, respectively. PCR was carried out in a 50 µL reaction mixture containing 2 µL DNA; 2 µL of 10 µM primer, both forward and reverse (Table 2); 25 µL of Taq DNA polymerase mix; and 19 µL double-distilled water. The PCR amplifications were carried out with the following cycling parameters: initial denaturation at 94 °C for 3 min, followed by 33 cycles of 30 s at 94 °C, annealing at a suitable temperature for 30 s for different loci: 55 °C for ITS, 52.4 °C for *LSU*, 53 °C for *SSU* and *RPB2*, 59.5 °C for *GAPDH*, 60.5 °C for *Alt a 1*, extension at 72 °C for 30 s, and a final elongation at 72 °C for 10 min. The cycling parameter for *TEF1-α* was as follows: 94 °C for 5 min; 40 cycles at 94 °C for 30 s; 59 °C for 30 s; 72 °C for 45 s; and a final extension at 72 °C for 7 min. The PCR products were sequenced at Sangon Biotech Co., Ltd. (Nanjing, China). 

### 4.5. Multigene Phylogenetic Analyses

The obtained sequences were analyzed using BLAST (https://www.ncbi.nlm.nih.gov/, accessed on 2 August 2023) to retrieve sequences with high similarities to the query sequences. Seventy-one isolates of 45 *Alternaria* species were obtained from GenBank for phylogenetic use in analyses (Table 3). The sequences of each gene/region were aligned with MAFFT ver. 7.313 (https://mafft.cbrc.jp/alignment/server, accessed on 2 August 2023) and manually adjusted using BioEdit ver. 7.0 [70,71]. The seven genes/region were concatenated by PhyloSuite ver. 7.313 [72]. ModelFinder was used to select the best-fit model, and phylogenetic relationships were inferred using maximum-likelihood (ML) analysis in IQtree ver. 1.6.8 and Bayesian inference (BI) in MrBayes 3.2.6 [73,74,75]. The ML analysis used the best model: GTR + I + G4 + F, with 1000 bootstrap replicates. The BI analysis used the best model: SYM + I + G, with 2 parallel runs and 2,000,000 generations, discarding the initial 25% of sampled data as burn-in. Phylogenetic trees were visualized using FigTree ver. 1.4.3 (https://tree.bio.ed.ac.uk/software/figtree/, accessed on 2 August 2023).

## 5. Conclusions

In the present study, we conducted fungal pathogen isolation and pathogenicity tests. We identified the nine fungal isolates (B1–B9) obtained, which are causing leaf blotch of *C. julianae*, based on multi-locus phylogenetic analyses using loci of ITS, *LSU*, *SSU*, *GAPDH*, *Alt a 1*, *RPB2*, *TEF1-α*, and morphological characteristics. The isolates were determined to be *A. arborescens* and *A. italica* within the *Alternaria* section *Alternaria*. To the best of our knowledge, this is the first report of *A. arborescens* and *A. italica* causing leaf blotch of *C. julianae* in either China or the wider world. The results of the study provide imperative and fundamental information for understanding the disease and performing future studies on the fungi/pathogens and the disease from mycological and phytopathological aspects. It is the first step in advancing the management of the disease.

## Figures and Tables

**Table 1:** Fungi isolated from diseased leaves of *Celtis julianae*.

Month	Number of Tissues	Number of Colonies		
		*Nigrospora* sp.	*Alternaria* sp.	*Nothophoma* sp.
September	100	50 (63%)	19 (24%)	10 (13%)
October	100	47 (54%)	33 (38%)	7 (8%)

**Table 2:** Primers used for PCR amplification in molecular identification of nine isolates (B1–B9).

Locus	Primers	Primer Sequences (5′-3′)	Advantages and Limitations	Reference
ITS	ITS1	TCCGTAGGTGAACCTGCGC	Universal fungal barcode, contains greater sequence variation, evolves faster, intragenomic variation gives the slow homogenization among the various copies	[64]
	ITS4	TCCTCCGCTTATTGATATGC		
*LSU*	LROR	ACCCGCTGAACTTAAGC	Conserved and variable domain, low rate of molecular evolution reduces the taxonomic resolution at the species-level	[65]
	LR5	TCCTGAGGGAAACTTCG		
*SSU*	NS1	GTAGTCATATGCTTGTCTC		[64]
	NS4	CTTCCGTCAATTCCTTTAAG		
*GAPDH*	GPD1	CAACGGCTTCGGTCGCATTG	Highly effective for heterologous protein expression in microorganisms, the expression level may increase under inducing treatments	[66]
	GPD2	GCCAAGCAGTTGGTTGTGC		
*Alt a 1*	Alt-al-for	ATGCAGTTCACCACCATCGC	A gene for the *Alternaria* major allergen, supports grouping of *Alternaria* spp. and related taxa	[67]
	Alt-a1-rev	ACGAGGGTGAYGTAGGCGTC		
*RPB2*	RPB2-5F2	GGGGWGAYCAGAAGAAGGC	Recover well-supported clades at shallow and deep taxonomic levels and has a better species-resolving power than rDNA markers	[68]
	RPB2-7cR	CCCATRGCT TGT YYRCCCAT		
*TEF1-α*	EF1-728F	CATCGAGAAGTTCGAGAAGG	Recover some deep and ordinal-level relationships but with greater branch support from nucleotides	[69]
	EF1-986R	TACTTGAAGGAACCCTTACC		

**Table 3:** Isolates of *Alternaria* spp. used in this study and corresponding GenBank accession numbers.

Species	Isolate	Locality, Host/Substrate	Accession Numbers						
			ITS	*LSU*	*SSU*	*GAPDH*	*Alt a 1*	*RPB2*	*TEF1-α*
*Alternaria alstroemeriae*	CBS 118808	USA, *Alstroemeria* sp.	KP124296	KP124447	KP124917	KP124153	KP123845	KP124764	KP125071
*A. alternantherae*	CBS 124392	China, *Solanum melongena*	KC584179	KC584251	KC584506	KC584096	KP123846	KC584374	KC584633
***A. alternata***	**CBS 916.96**	**India, *Arachis hypogaea***	**AF347031**	**DQ678082**	**KC584507**	**AY278808**	**AY563301**	**KC584375**	**KC584634**
*A. alternata*	CBS 112249	-, -	KP124338	KP124490	KP124960	KP124192	KP123886	KP124806	KP125114
***A. arborescens***	**CBS 102605**	**USA, *Solanum lycopersicum***	**AF347033**	**KC584253**	**KC584509**	**AY278810**	**AY563303**	**KC584377**	**KC584636**
*A. arborescens*	B1 = CFCC 59038 *	China, *Celtis julianae*	OQ691659	OQ692430	OQ692438	OQ710109	OQ710107	OQ710111	OQ710113
*A. arborescens*	B2 = CFCC 59039 *	China, *Celtis julianae*	OQ691640	OQ692431	OQ692439	OQ710110	OQ710108	OQ710112	OQ710114
*A. arborescens*	B7 *	China, *Celtis julianae*	OR243734	OR366490	OR366484	OR475216	OR475232	OR475224	OR475210
*A. arborescens*	B8 *	China, *Celtis julianae*	OR243735	OR366491	OR366485	OR475217	OR475231	OR475225	OR475211
*A. arborescens*	B9 *	China, *Celtis julianae*	OR243736	OR366492	OR366486	OR475218	OR475233	OR475226	OR475212
*A. arborescens*	CBS 119545	New Zealand, *Senecio skirrhodon*	KP124409	KP124562	KP125032	KP124260	KP123956	KP124879	KP125187
***A. arctoseptata***	**MFLUCC 21-0139**	**Italy, *Lathyrus* sp. (Fabaceae)**	**-**	**MZ621948**	**MZ621874**	**0K236608**	**OK236755**	**OK236655**	**OK236702**
***A. avenicola***	**CBS 121459**	**Norway, *Avena* sp.**	**KC584183**	**KC584256**	**KC584512**	**KC584100**	-	**KC584380**	**KC584639**
***A. axiaeriisporifera***	**CBS 118715**	**New Zealand, *Gypsophila paniculata***	**KC584184**	**KC584257**	**KC584513**	**KC584101**	-	**KC584381**	**KC584640**
***A. baoshanensis***	**MFLUCC 21-0124**	**China, *Curcubita moschata***	**MZ622003**	**MZ621952**	**MZ621878**	**OK236613**	**OK236760**	**OK236659**	**OK236706**
***A. betae-kenyensis***	**CBS 118810**	**Kenya, *Beta vulgaris* var. *cicla***	**KP124419**	**KP124572**	**KP125042**	**KP124270**	**KP123966**	**KP124888**	**KP125197**
*A. brassicae*	CBS 116528	USA, *Brassica oleracea*	KC584185	KC584258	KC584514	KC584102	-	KC584382	KC584641
*A. brassicicola*	CBS 118699	USA, *Brassica oleracea*	JX499031	KC584259	KC584515	KC584103	**-**	KC584383	KC584642
***A. breviconidiophora***	**MFLUCC 22-0075**	**Italy, *Digitalis* sp.** **(Scrophulariaceae)**	**MZ621997**	**MZ621944**	**MZ621870**	**OK236604**	**OK236751**	**OK236651**	**OK236698**
***A. burnsii***	**CBS 107.38**	**India, *Cuminum cyminum***	**KP124420**	**KP124573**	**KP125043**	**JQ646305**	**KP123967**	**KP124889**	**KP125198**
***A. carotiincultae***	**CBS 109381**	**USA, *Daucus carota***	**KC584188**	**KC584262**	**KC584518**	**KC584106**	-	**KC584386**	**KC584645**
*A. cinerariae*	CBS 116495	USA, *Ligularia* sp.	KC584190	KC584265	KC584521	KC584109	**-**	KC584389	KC584648
*A. conoidea*	CBS 132.89	Saudi Arabia, *Ricinus communis*	FJ348226	KC584327	KC584585	FJ348227	FJ348228	KC584452	KC584711
*A. dauci*	CBS 117097	USA, *Daucus carota*	KC584192	KC584268	KC584524	KC584111	KJ718678	KC584392	KC584651
***A. doliconidium***	**KUN-HKAS 100840T**	**Italy, *Rosa canina***	**NR158361**	**NG069551**	**NG065142**	**-**	**-**	**-**	**-**
***A. eichhorniae***	**CBS 489.92**	**India, *Eichhornia crassipes***	**KC146356**	**KP124579**	**KP125049**	**KP124276**	**KP123973**	**KP124895**	**KP125204**
***A. ellipsoidea***	**CBS 119674**	**USA, *Dianthus barbatus***	**KC584196**	**KC584272**	**KC584528**	**KC584115**	-	**KC584396**	**KC584655**
***A. ellipsoidialis***	**MFLUCC 21-0132**	**Italy, *Brassica* sp. (Brassicaceae)**	**MZ621989**	**MZ621936**	**MZ621862**	**OK236596**	**OK236743**	**OK236643**	**OK236690**
***A. eupatoriicola***	**MFLUCC 21-0122**	**Italy, *Eupatorium cannabinum*** **(Asteraceae)**	**MZ621982**	**MZ621929**	**MZ621855**	**OK236589**	**OK236736**	**OK236636**	**OK236683**
***A. falcata***	**MFLUCC 21-0123**	**Italy, *Atriplex* sp.** **(Chenopodiaceae)**	**MZ621992**	**MZ62139**	**MZ621865**	**OK236599**	**OK236746**	**OK236649**	**OK236693**
*A. gaisen*	CBS 118488	Japan, *Pyrus pyrifolia*	KP124427	KP124581	KP125051	KP124278	KP123975	KP124897	KP125206
***A. gossypina***	**CBS 104.32**	**Zimbabwe, *Gossypium* sp.**	**KP124430**	**KP124584**	**KP125054**	**JQ646312**	**JQ646395**	**KP124900**	**KP125209**
***A. gypsophilae***	**CBS 107.41**	**Netherlands, *Gypsophila elegans***	**KC584199**	**KC584277**	**KC584533**	**KC584118**	**KJ718688**	**KC584401**	**KC584660**
*A. helianthiinficiens*	CBS 117370	UK, *Helianthus annuus*	KC584200	KC584278	KC584534	KC584119	**-**	KC584402	KC584661
***A. helianthiinficiens***	**CBS 208.86**	**USA, *Helianthus annuus***	**JX101649**	**KC584279**	**KC584535**	**KC584120**	-	**KC584403**	**EU130548**
***A. infectoria***	**CBS 210.86**	**UK, *Triticum aestivum***	**DQ323697**	**KC584280**	**KC584536**	**AY278793**	**FJ266502**	**KC584404**	**KC584662**
***A. iridiaustralis***	**CBS 118486**	**Australia, *Iris* sp.**	**KP124435**	**KP124589**	**KP125059**	**KP124284**	**KP123981**	**KP124905**	**KP125214**
***A. italica***	**MFLUCC 14-0421T**	**Italy, Pleosporaceae**	**MG764017**	**MG818319**	**-**	**-**	**-**	**MG859737**	**-**
*A. italica*	B3 = CFCC 59359 *	China, *Celtis julianae*	OR272062	OR366487	OR366480	OR475213	OR475227	OR475220	OR475207
*A. italica*	B4 = CFCC 59309 *	China, *Celtis julianae*	OR243731	OR366526	OR366481	OR475219	OR475228	OR475221	OR250485
*A. italica*	B5 = CFCC 59310 *	China, *Celtis julianae*	OR243732	OR366488	OR366482	OR475214	OR475229	OR475222	OR475208
*A. italica*	B6 = CFCC 59311 *	China, *Celtis julianae*	OR243733	OR366489	OR366483	OR475215	OR475230	OR475223	OR475209
***A. jacinthicola***	**CBS 133751**	**Mali, *Eichhornia crassipes***	**KP124438**	**KP124592**	**KP125062**	**KP124287**	**KP123984**	**KP124908**	**KP125217**
***A. juxtiseptata***	**CBS 119673**	**Australia, *Gypsophila paniculata***	**KC584202**	**KC584282**	**KC584538**	**KC584122**	-	**KC584406**	**KC584664**
***A. lathyri***	**MFLUCC 21-0140**	**Italy, *Lathyrus* sp. (Fabaceae)**	**MZ621974**	**MZ621921**	**MZ621847**	**OK236581**	**OK236728**	**OK236628**	**OK236675**
*A. longipes*	CBS 540.94	USA, *Nicotiana tabacum*	AY278835	KC584285	KC584541	AY278811	AY563304	KC584409	KC584667
***A. macroconidia***	**MFLUCC 21-0134**	**Italy, *Spartium junceum***	**MZ622001**	**MZ621950**	**MZ621876**	**OK236610**	**OK236757**	**OK236657**	**OK236704**
***A. macrospora***	**CBS 117228**	**USA, *Gossypium barbadense***	**KC584204**	**KC584286**	**KC584542**	**KC584124**	**KJ718702**	**KC584410**	**KC584668**
***A. mimicula***	**CBS 118696**	**USA, *Lycopersicon esculentum***	**FJ266477**	**KC584287**	**KC584543**	**AY562415**	**GQ180094**	**KC584411**	**KC584669**
***A. minimispora***	**MFLUCC 21-0127**	**Thailand, *Citrullus lanatus***	**MZ621980**	**MZ621927**	**MZ621853**	**OK236587**	**OK236734**	**OK236634**	**OK236681**
***A. muriformispora***	**MFLUCC 22-0073**	**Italy, *Plantago* sp. (Plantaginaceae)**	**MZ621976**	**MZ621923**	**MZ621849**	**OK236583**	**OK236730**	**OK236630**	**OK236677**
*A. nobilis*	CBS 116490	New Zealand, *Dianthus caryophyllus*	KC584208	KC584291	KC584547	KC584127	**-**	KC584415	KC584673
***A. oblongoellipsoidea***	**MFLUCC 22-0074**	**Italy, *Cichorium intybus***	**MZ621967**	**MZ621914**	**MZ621840**	**OK236574**	**OK236721**	**OK236621**	**OK236668**
***A. obpyriconidia***	**MFLUCC 21-0121**	**Italy, *Vicia faba***	**MZ621978**	**MZ621925**	**MZ621851**	**OK236585**	**OK236732**	**OK236633**	**OK236680**
***A. orobanches***	**MFLUCC 21-0137**	**Italy, *Orobanche* sp.**	**MZ622007**	**MZ621956**	**MZ621882**	**-**	**OK236763**	**-**	**OK236710**
***A. ovoidea***	**MFLUCC 14-0427**	**Italy, *Dactylis glomerata***	**MZ622005**	**MZ621954**	**MZ621880**	**OK236614**	**OK236761**	**OK236661**	**OK236708**
***A. perpunctulata***	**CBS 115267**	**USA, *Alternanthera philoxeroides***	**KC584210**	**KC584294**	**KC584550**	**KC584129**	**JQ905111**	**KC584418**	**KC584676**
***A. petroselini***	**CBS 112.41**	**–, *Petroselinum sativum***	**KC584211**	**KC584295**	**KC584551**	**KC584130**	-	**KC584419**	**KC584677**
***A. phragmiticola***	**MFLUCC 21-0125**	**Italy, *Phragmites* sp.**	**MZ621994**	**MZ621941**	**MZ621867**	**OK236602**	**OK236749**	**OK236649**	**OK236696**
*A. porri*	CBS 116698	USA, *Allium cepa*	DQ323700	KC584297	KC584553	KC584132	KJ718726	KC584421	KC584679
***A. pseudoinfectoria***	**MFLUCC 21-0126**	**Italy, *Chenopodium* sp.**	**MZ621984**	**MZ621931**	**MZ621857**	**OK236591**	**OK236738**	**OK236638**	**OK236685**
***A. pseudorostrata***	**CBS 119411**	**USA, *Euphorbia pulcherrima***	**JN383483**	**KC584298**	**KC584554**	**AY562406**	**AY563295**	**KC584422**	**KC584680**
***A. radicina***	**CBS 245.67**	**USA, *Daucus carota***	**KC584213**	**KC584299**	**KC584555**	**KC584133**	**FN689405**	**KC584423**	**KC584681**
***A. rostroconidia***	**MFLUCC 21-0136**	**Italy, *Arabis* sp.**	**MZ621969**	**MZ621916**	**MZ621842**	**OK236576**	**OK236723**	**OK236623**	**OK236670**
***A. salicicola***	**MFLUCC 22-0072**	**Russia, *Salix alba***	**MZ621999**	**MZ621946**	**MZ621872**	**OK236606**	**OK236753**	**OK236653**	**OK236700**
*A. saponariae*	CBS 116492	USA, *Saponaria officinalis*	KC584215	KC584301	KC584557	KC584135	**-**	KC584425	KC584683
***A. selini***	**CBS 109382**	**Saudi Arabia, *Petroselinum crispum***	**AF229455**	**KC584302**	**KC584558**	**AY278800**	**FJ266504**	**KC584426**	**KC584684**
***A. septorioides***	**CBS 106.41**	**Netherlands, *Reseda odorata***	**KC584216**	**KC584303**	**KC584559**	**KC584136**	-	**KC584427**	**KC584685**
***A. setosa***	**YZU 191101**	**China, *Iris japonica***	**OP2341770**	**-**	**-**	**OP352306**	**OP352294**	**OP352294**	**OP374459**
*A. smyrnii*	CBS 109380	UK, *Smyrnium olusatrum*	AF229456	KC584305	KC584561	KC584138	**-**	KC584429	KC584687
*A. solani*	CBS 116651	USA, *Solanum tuberosum*	KC584217	KC584306	KC584562	KC584139	GQ180097	KC584430	KC584688
***A. solidaccana***	**CBS 118698**	**Bangladesh, Soil**	**KC584219**	**KC584308**	**KC584564**	**KC584141**	-	**KC584432**	**KC584690**
*A. sonchi*	CBS 119675	Canada, *Sonchus asper*	KC584220	KC584309	KC584565	KC584142	-	KC584433	KC584691
***A. tectorum***	**YZU 161050**	**China, *Iris tectorum***	**OP341728**	**-**	**-**	**OP352303**	**OP293714**	**OP352291**	**OP374456**
*A. tectorum*	YZU 161052	**China, *Iris tectorum***	-	-	OP341817.1	OP352304.1	OP293715.1	OP352292.1	OP374457.1
*A. tomato*	CBS 103.30	Unknown, *Solanum lycopersicum*	KP124445	KP124599	KP125069	KP124294	KP123991	KP124915	KP125224
***A. torilis***	**MFLUCC 14-0433**	**Italy, *Torilis arvensis***	**MZ621988**	**MZ621935**	**MZ621861**	**OK236594**	**OK236741**	**OK236641**	**OK236688**
*A. vaccariae*	CBS 116533	USA, *Vaccaria hispanica*	KC584223	KC584314	KC584570	KC584146	JQ646386	KC584438	KC584696
***A. vaccariicola***	**CBS 118714**	**USA, *Vaccaria hispanica***	**KC584224**	**KC584315**	**KC584571**	**KC584147**	**JQ646384**	**KC584439**	**KC584697**
***A. vitis***	**MFLUCC 17-1109T**	**Chile, China, El Salvador, Greece, India, Italy, Romania, Russia, Thailand, Turkmenistan, Pleosporaceae**	**MG764007**	**-**	**-**	**-**	**-**	**-**	**-**

* Isolates in this study. Ex-type cultures are shown in bold. Abbreviations: CBS: Culture collection of the Westerdijk Fungal Biodiversity Institute, Utrecht, The Netherlands; CFCC: China Forestry Culture Collection Center, Beijing, China; KUN-HKAS: Herbarium of Cryptogams Kunming Institute of Botany Academia Sinica, Yunnan, China; MFLUCC: Mae Fah Luang University Culture Collection, Chiang Rai, Thailand; YZU: Fungal Herbarium of Yangtze University, Jingzhou, Hubei, China.

## Data Availability

All data generated or analyzed during this study are included in this article.
